# Sub-nanometre quality X-ray mirrors created using ion beam figuring

**DOI:** 10.1107/S1600577524004594

**Published:** 2024-06-21

**Authors:** Arindam Majhi, Riley Shurvinton, Paresh Chandra Pradhan, Matthew Hand, Weichen Gu, Murilo Bazan Da Silva, Simone Moriconi, Ioana Nistea, Simon G. Alcock, Hongchang Wang, Kawal Sawhney

**Affiliations:** a Diamond Light Source Harwell Science and Innovation Campus DidcotOX11 0DE United Kingdom; b MOE Key Laboratory of Advanced Micro-Structured Materials, Institute of Precision Optical Engineering, School of Physics Science and Engineering Tongji University Shanghai200092 People’s Republic of China; DESY, Germany

**Keywords:** ion beam figuring (IBF), height error and slope error, X-ray optics, silicon optics, deterministic figuring

## Abstract

Ion beam figuring (IBF) is a powerful technique for figure correction of X-ray mirrors to a high accuracy. Here, recent technical advancements in the IBF instrument developed at Diamond Light Source are presented and experimental results for figuring of X-ray mirrors are given. The IBF system is equipped with a stable DC gridded ion source (120 mm diameter), a four-axis motion stage to manipulate the optic, a Faraday cup to monitor the ion-beam current, and a camera for alignment. A novel laser speckle angular measurement instrument also provides on-board metrology. To demonstrate the IBF system’s capabilities, two silicon X-ray mirrors were processed. For 1D correction, a height error of 0.08 nm r.m.s. and a slope error of 44 nrad r.m.s. were achieved. For 2D correction over a 67 mm × 17 mm clear aperture, a height error of 0.8 nm r.m.s. and a slope error of 230 nrad r.m.s. were obtained. For the 1D case, this optical quality is comparable with the highest-grade, commercially available, X-ray optics.

## Abstract 2

Recent advancements are presented for an ion beam figuring system developed at Diamond Light Source, demonstrating surface correction of X-ray mirrors to achieve sub-nanometre r.m.s. height errors and <50 nrad r.m.s. slope errors.

## 1. Introduction

In recent times, the demand for high-quality X-ray mirrors has rapidly increased across various scientific and technological fields, including modern synchrotron sources (Hu *et al.*, 2022 ▸; Wang, Huang, Ke *et al.*, 2023 ▸), free-electron lasers (Siewert *et al.*, 2014 ▸) and astronomical telescopes (Ghigo *et al.*, 2014 ▸; Kim *et al.*, 2021 ▸). To meet the demands for nano-focusing, coherence preservation and exceptional energy resolution (Mimura *et al.*, 2007 ▸; Hignette *et al.*, 2005 ▸; Yumoto *et al.*, 2013 ▸), such mirrors typically require height errors of the order of 1 nm or better, and slope errors of 100 nrad r.m.s. or better. Achieving such exceptional figure accuracy is challenging for traditional manufacturing techniques such as mechanical polishing, especially for curved or aspheric profiles. This motivates the investigation of deterministic, high-precision figuring methods guided by accurate metrology data (Idir *et al.*, 2015 ▸; Wang *et al.*, 2019 ▸; Zhou *et al.*, 2016 ▸).

Ion beam figuring (IBF) (Allen & Romig, 1990 ▸; Carnal *et al.*, 1992 ▸; Xie & Li, 2013 ▸; Demmler *et al.*, 2010 ▸) has emerged as a prominent choice for accurate correction of optical surfaces. IBF is a non-contact, computer-controlled optical surfacing (CCOS) method (Cheng, 2016 ▸), which utilizes motorized stages and a carefully controlled ion source to figure a mirror surface to the required accuracy.

In IBF, energetic ions (typically argon) bombard the surface of a mirror to selectively remove material at the target location. Deterministic correction is achieved by varying the motion of the ion beam over the mirror’s surface. At appropriate ion energies, material can be removed without degrading the surface micro-roughness. This allows the figure error of the mirror to be corrected whilst still maintaining excellent micro-roughness, which is crucial for X-ray performance.

IBF is gaining popularity due to its excellent precision, high predictability, and linearity of removal. It has been effectively employed to create both curved and flat mirrors, achieving surface profiles with accuracy at the nanometre scale to meet the demanding tolerances required for X-ray optic applications (Wang, Huang, Zhu *et al.*, 2023 ▸; Wang, Huang, Ke *et al.*, 2023 ▸). However, attaining this accuracy depends on high-quality metrology of the optical surface and precise alignment of the substrate relative to the ion beam. Several iterations of measurement and correction are typically required to converge to the desired optical quality.

In-house IBF plants at synchrotron and free-electron laser laboratories have been seeing particular interest in recent years. Due to the reliance of the IBF process on precise metrology, laboratories with existing metrology facilities can take advantage of them to measure the surface of a mirror before and after IBF. This allows an in-house IBF process to converge quickly and efficiently to the required figure accuracy. To this end, the Optics & Metrology and Engineering groups at Diamond Light Source (DLS) have recently designed, built and commissioned an in-house IBF system (Hand *et al.*, 2019 ▸).

A major disadvantage of many IBF systems is that the mirror needs to be removed from the vacuum chamber after every iteration of the IBF process to measure the new surface profile. Loading/unloading the sample, and venting/pumping the vacuum vessel for each cycle of IBF and metrology can be a time-consuming activity. To circumvent this, a novel on-board metrology device was added to the system: a laser speckle angular measurement (SAM) instrument (Wang, Moriconi *et al.*, 2021 ▸). The SAM can be used to measure the curvature and slope of the mirror, allowing fast and efficient metrology without needing to remove the sample. This allows the removal function of the ion beam to be determined using on-board measurements.

In this paper, we demonstrate the recent achievements of the DLS IBF system, including examples of 1D and 2D correction of silicon X-ray mirrors. In Section 2, we describe the instrumentation of the IBF system, including surface metrology using the laser-based SAM instrument and a laser-based Fizeau interferometer. Optimization of the IBF process parameters and sample alignment are presented in Section 3. In Section 4, the robust iterative Fourier transform-based dwell time algorithm (RIFTA) (Wang *et al.*, 2020 ▸) is employed to derive the optimal dwell time map to correct a given surface. Finally, the results of the optical figuring process are presented in Section 5, beginning with 1D correction, and concluding with the most recent 2D correction results, demonstrating the achievement of sub-nanometre error correction.

## 2. Instrumentation

### 2.1. IBF hardware

The in-house IBF system (Hand *et al.*, 2019 ▸) at Diamond is housed within a stainless-steel vacuum chamber measuring ∼1.6 m in length, 0.9 m in width and 1.0 m in height. Key components include a four-axis motion stage; a stationary, large-diameter DC gridded ion source; a CCD camera and imaging lens; and the SAM metrology instrument. The mirror under figuring is mounted on the motion stage, which moves along three translational axes and one rotational axis, while the ion source is fixed. During IBF, the vessel is pumped down to a base pressure of ∼1 × 10^−6^ mbar, while the working pressure is ∼3 × 10^−4^ mbar when the ion source is in operation.

The KDC100 ion source (Kaufman & Robinson Inc.) emits a 120 mm-diameter ion beam, which is slightly divergent. The source uses argon (Ar) gas with a purity of >99.999%, supplied through a mass flow controller at a constant and stable rate of 7.0 standard cubic centimeters per minute (s.c.c.m.). An external neutralizer located in front of the ion source supplies electrons to neutralize the space charge of the ion beam and ensure uniform current density. The shape and size of the ion beam that reaches the optic is controlled using two pyrolytic graphite aperture plates, located after the neutralizer. The first plate has an aperture allowing a central 20 mm × 10 mm section of the ion beam to pass through. A second plate further refines the beam shape by allowing it to pass through one of several smaller apertures of varying shape (circular or rectangular) and dimension (up to 10 mm). The second plate is mounted on a linear translation stage which lets it move in front of the beam, allowing the beam size to be changed as desired.

**Figure 1:**
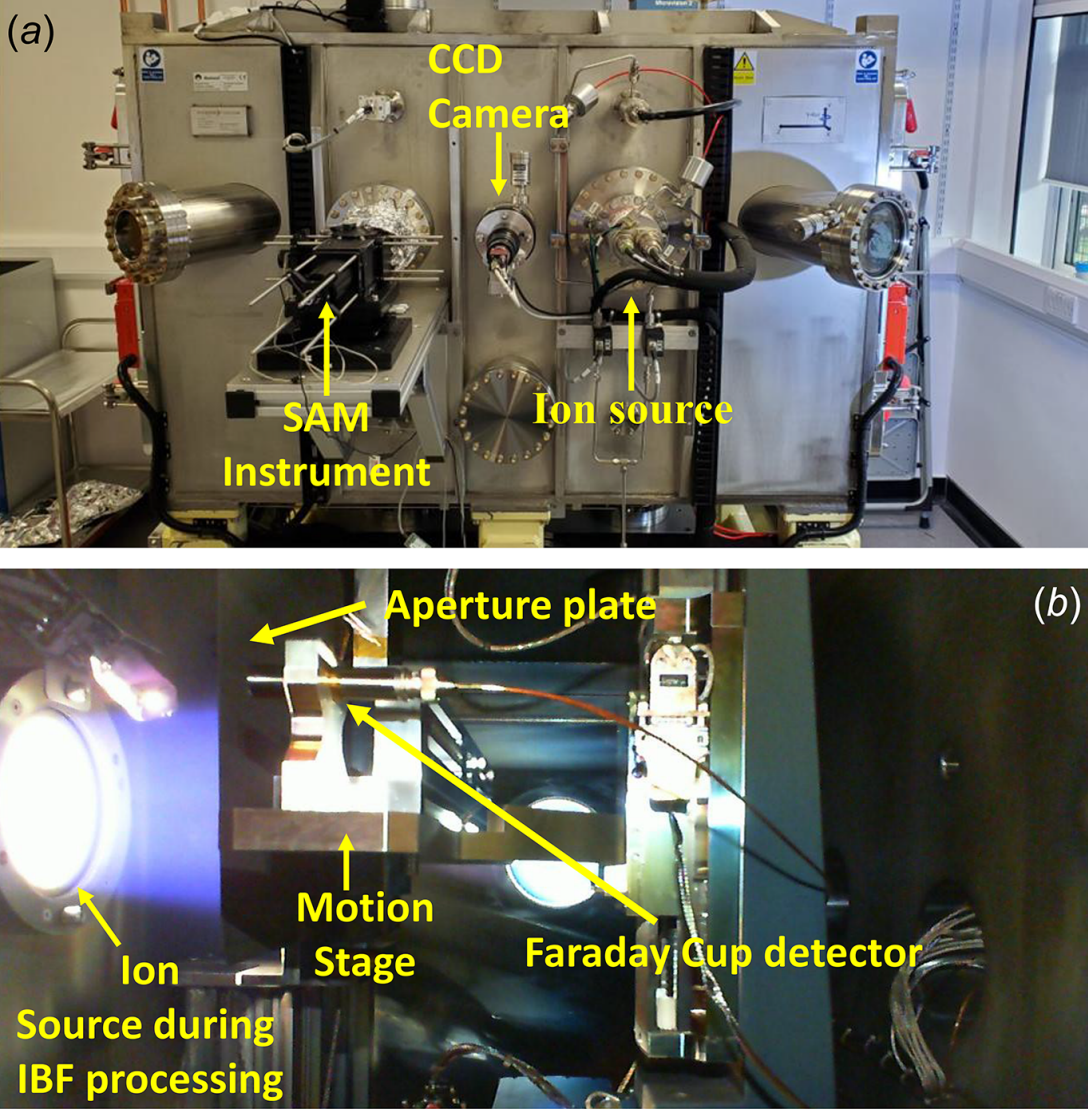
Photographs of the IBF system. (*a*) Exterior view of the vacuum chamber, with labelling of important components (SAM instrument, camera and ion source). (*b*) Side view inside the chamber during figuring, showing the KRi KDC100 ion source and neutralizer, the aperture plates and the motion stage, including a mounted Faraday cup which is used to align the ion beam relative to the motion system.

A programmable logic controller (PLC) manages the pumping and venting of the vessel, and controls the safety interlocks for the motion stages and the ion source. The motion stages are controlled using Diamond’s GeoBrick LV (Delta Tau) motion controller, which can execute provided position-velocity-time motion data. The travel range of the motion stages allows an area of ∼300 mm × 50 mm to be corrected by the ion beam. The ion source and neutralizer are controlled by KRI ion source controller modules, offering both local and remote control of electrical and gas flow parameters. Furthermore, Python scripts have been incorporated to manage system control. Photographs of the IBF system are shown in Fig. 1 ▸.

### 2.2. On-board metrology with SAM

**Figure 2:**
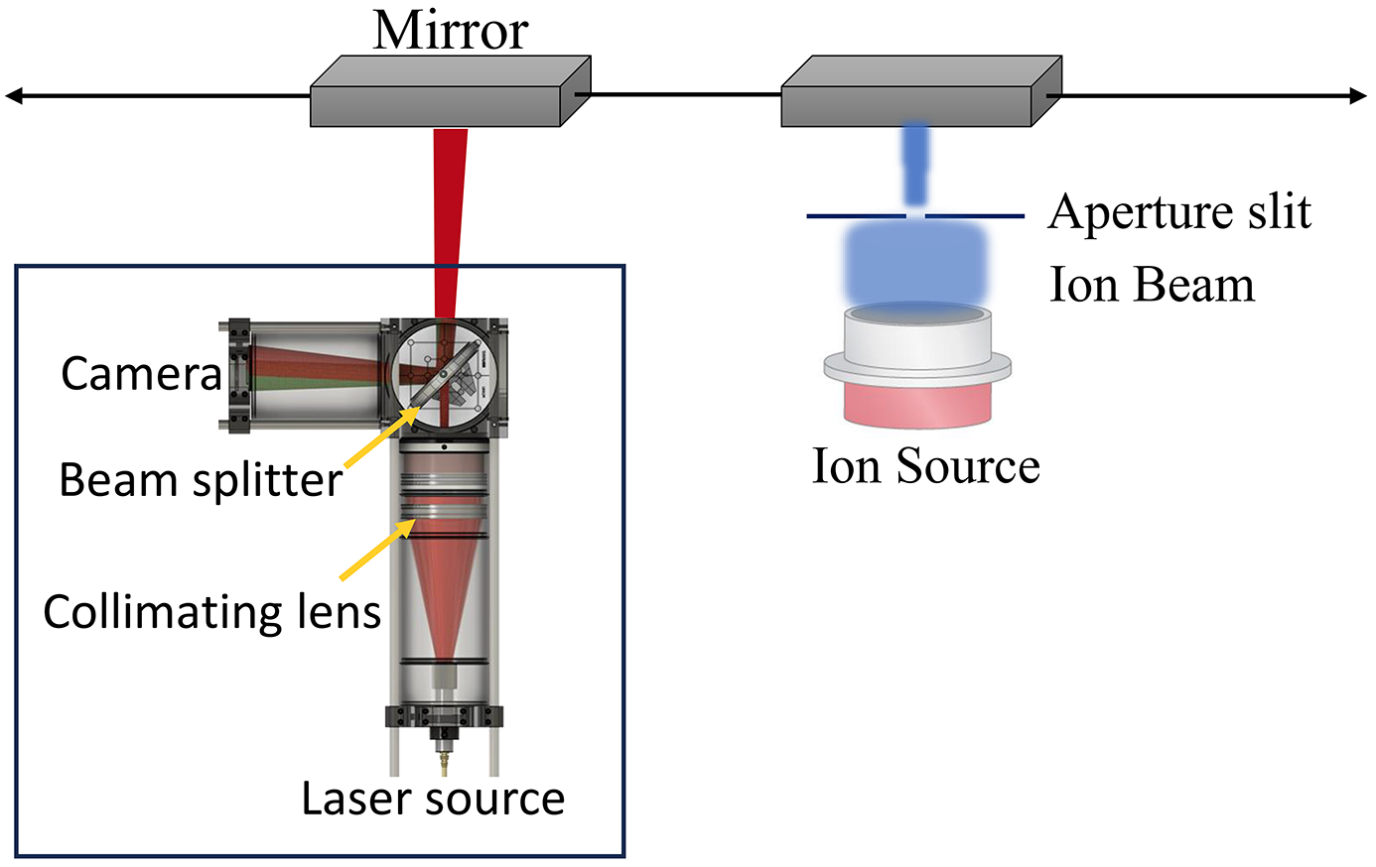
Schematic depiction of the SAM instrument, including labelling of the major components with figuring process.

The inclusion of an on-board SAM (Wang, Moriconi *et al.*, 2021 ▸) metrology instrument in the IBF system provides several significant benefits. SAM is a compact and low-cost instrument, which enables measurement of the local inverse radius of curvature or slope of a mirror. The instrument records images of a reflected speckle pattern at different points on the mirror surface. An algorithm tracks the displacement of the speckle pattern between images to calculate the curvature of the mirror. SAM provides fast metrology feedback after each figuring iteration, which significantly reduces the overall time needed. It can also be used to measure the depth of etched ion beam craters, allowing the beam removal function to be determined. A schematic representation of SAM is shown in Fig. 2 ▸.

**Figure 3:**
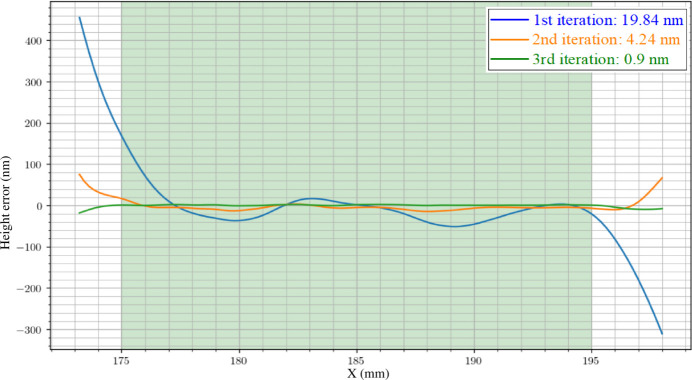
SAM measurements of an Si mirror for 1D-IBF correction, demonstrating the improvement in r.m.s. height error using the SAM instrument.

The initial results demonstrating 1D IBF correction applied to a test Si mirror using SAM instrument. In Fig. 3 ▸, the residual r.m.s. height error undergoes improvement throughout the figuring process, as measured by the on-board SAM. The r.m.s. height error is notably reduced from 20 nm to 0.9 nm within the active region after the third iteration of IBF correction.

### 2.3. Surface metrology using Fizeau interferometry

*Ex situ* metrology of the X-ray mirrors was performed in the Optics Metrology Laboratory using a stitching interferometry system developed in-house (Da Silva *et al.*, 2023 ▸). The system uses a four-axis motion stage (enabling pitch and roll rotation, and horizontal and vertical translation of the optic) and a Zygo Verifire HDX Fizeau interferometer. The interferometer produces a laser beam 150 mm in diameter and can image a range of optical surfaces at a lateral resolution of ∼46 µm and a CCD pixel count of 3392 × 3392. The Fizeau stitching system exhibits slope error repeatability levels of <15 nrad r.m.s., allowing for meticulous comparison between stitched maps. Python code is used to control the data acquisition, by automating the motion of the stage and measurement and saving of data by the interferometer.

Stitching interferometry allows a large optic to be measured by taking many smaller ‘sub-aperture’ measurements at different points on the optic’s surface. These overlapping sub-aperture measurements are then carefully combined (‘stitched’) to provide a composite image of the entire optical surface. However, to do so, the tip, tilt, piston and lateral displacement of each sub-aperture must be precisely aligned, which can be challenging and computationally expensive. To tackle this issue, a software tool called *PyLOSt* (*Python Large Optic Stitching*) is employed, which was primarily developed at the ESRF, France, as part of the MooNpics project (Adapa, 2020 ▸). *PyLOSt* offers a user-friendly interface within the Orange Data Mining framework (Demšar *et al.*, 2013 ▸), and calculates stitching of sub-apertures using a global optimization algorithm. The stitching algorithms in *PyLOSt* are compatible with both 1D and 2D measurements of either height or slope.

## 3. IBF process optimization and sample alignment

To ensure a well controlled deterministic figuring process, the performance of the ion source must be stable, and its removal of material must be precisely characterized. The stability of the ion source can be optimized by controlling multiple parameters including the gas flow rate, grid voltage and ion current. Adjusting the beam voltage controls the material removal rate and the size of the beam footprint. A higher removal rate and a narrower footprint are both advantageous for faster and more precise figuring.

**Figure 4:**
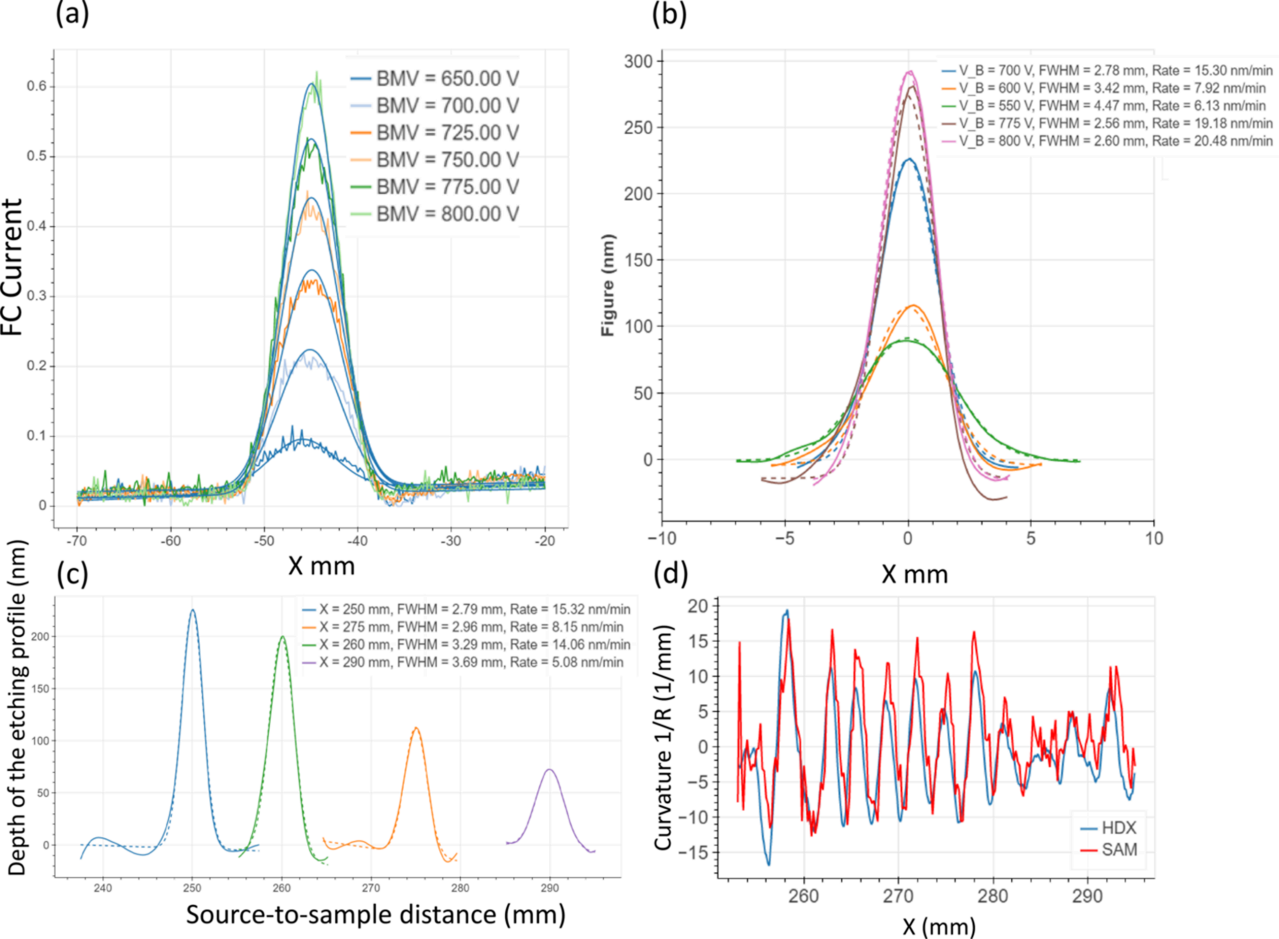
Optimization of the IBF process parameters. (*a*) Ion beam current profiles measured using a ‘Faraday cup’ intensity monitor, for a beam passing through a 1 mm × 8 mm aperture at beam voltages between 650 V and 800 V. Larger beam voltages yield an optimized profile for figuring. (*b*) Cross-sectional SAM measurements of craters etched on a Si wafer for beam voltages between 650 V and 800 V. Similarly to the Faraday cup measurements, a larger beam voltage gives an optimal removal profile. (*c*) Cross-sectional SAM measurements of craters etched on a Si wafer at a beam voltage of 800 V at different distances between the source and the sample. When the sample is closer to the source, the resulting crater is deeper and narrower, which is preferable for figuring. (*d*) A comparison between SAM and HDX measurements of the radius of curvature of a Si mirror over 50 mm. This indicates that the SAM gives good agreement with the HDX for coarse measurements.

The properties of the ion beam can be determined by measuring the depth and shape of an etched crater on an optic; or by monitoring the current profile of the ion beam using a Faraday cup. This can be used to assess the impact of beam voltage and source–sample distance on the beam removal function. Fig. 4 ▸(*a*) shows how varying the beam voltage affects the Faraday cup measurements of the beam profile. When the beam voltage increases from 650 V to 800 V, the resulting ion beam profile is narrower and has a higher peak current. Fig. 4 ▸(*b*) shows how the beam voltage affects the shape of etched craters on a Si wafer, measured using 1D SAM. Similarly to the measurements from the Faraday cup, the etched craters are narrower and have a greater depth at higher beam voltages, with the depth increasing from 85 nm at a voltage of 550 V to 290 nm at 800 V. From these results, the optimal beam voltage was set at 800 V to maximize removal rate.

Fig. 4 ▸(*c*) shows how the distance between the ion source and the sample surface affects the shape and size of etched craters, as measured by 1D SAM with a beam voltage of 800 V. The measurements of craters etched at 800 V for different source-to-sample distances, which demonstrates that a taller and narrower removal profile is also obtained when the sample is closer to the ion source. These results indicate that to optimize the removal profile and etching rate, it is necessary to maximize the voltage and minimize the distance between the surface under correction and the ion source. Finally, to validate the accuracy of the SAM measurements, *in situ* data were compared with *ex situ* Fizeau interferometry. Fig. 4 ▸(*d*) shows that both SAM and HDX give consistent results for slope measurement after figuring period structures into a silicon mirror.

The success of the figuring process relies on both accurate metrology and careful alignment between different coordinate systems. The position of the measured surface features in the metrology coordinates must be matched exactly to the position of the ion beam on the surface in the IBF coordinates, to ensure accurate removal of material. This is most often done using a feature, known as a ‘fiducial’, whose location can be determined in both coordinate systems to align between them.

**Figure 5:**
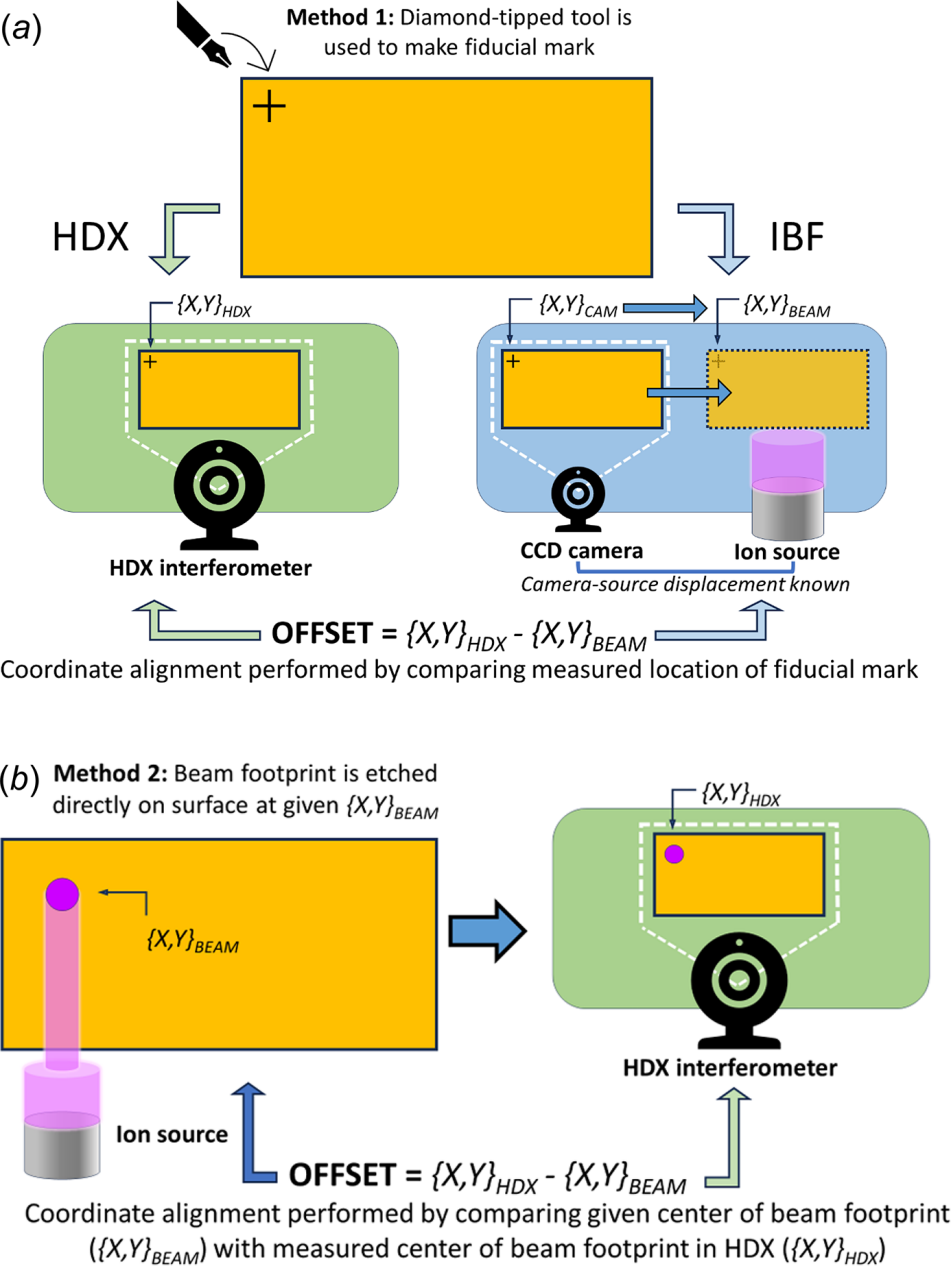
(*a*) Schematic representation of a fiducialization method (‘Method 1’) involving marking a cross on the sample surface using a diamond-tipped tool. The measured location of the mark in both the HDX and IBF is used to align between the two coordinate systems. (*b*) Schematic representation of a fiducialization method (‘Method 2’) involving etching a beam footprint on the sample at known IBF system coordinates. This is then compared with the measured centre of the beam footprint in the HDX (or SAM) coordinates to align the two coordinate systems.

A common approach is to etch a fiducial mark, usually a small cross, onto the optical surface (outside the clear aperture region) using a diamond-tipped tool or a laser marker. Fig. 5 ▸(*a*) shows a representation of this (‘Method 1’). An alternative method is to use the ion beam to etch one or more fiducial marks onto the sample, either craters (if the beam is kept stationary) or lines/grooves (if the beam is moving during etching). The centre of the etched footprint (crater or groove) is exactly known in the IBF coordinates, and it can be determined in the metrology coordinates by fitting an analytical function (*e.g* a 2D Gaussian or super-Gaussian for a circular footprint) to the measured surface data. A schematic of this approach (‘Method 2’) is shown in Fig. 5 ▸(*b*). This also allows exact measurement of the ion beam removal rate for the particular sample under figuring, as the removal rate may change slightly depending on sample history.

The action of an ion beam on a surface, *e.g* the distribution of material removal per unit time, is referred to as the beam removal function (BRF) (Idir *et al.*, 2015 ▸). It can be represented by a function *B*(*x*,*y*), where *B* is the removal rate (typically in nm s^−1^) and *x*, *y* represent the 2D area around the centre of the beam. The BRF can be obtained directly from measurement of an etched beam footprint; but, more commonly, it is assumed to have the shape of an analytical function, such as a 2D Gaussian or higher-order super-Gaussian for a beam generated from a circular aperture. The parameters of the function can then be extracted by fitting the appropriate function to the measured beam footprint.

**Figure 6:**
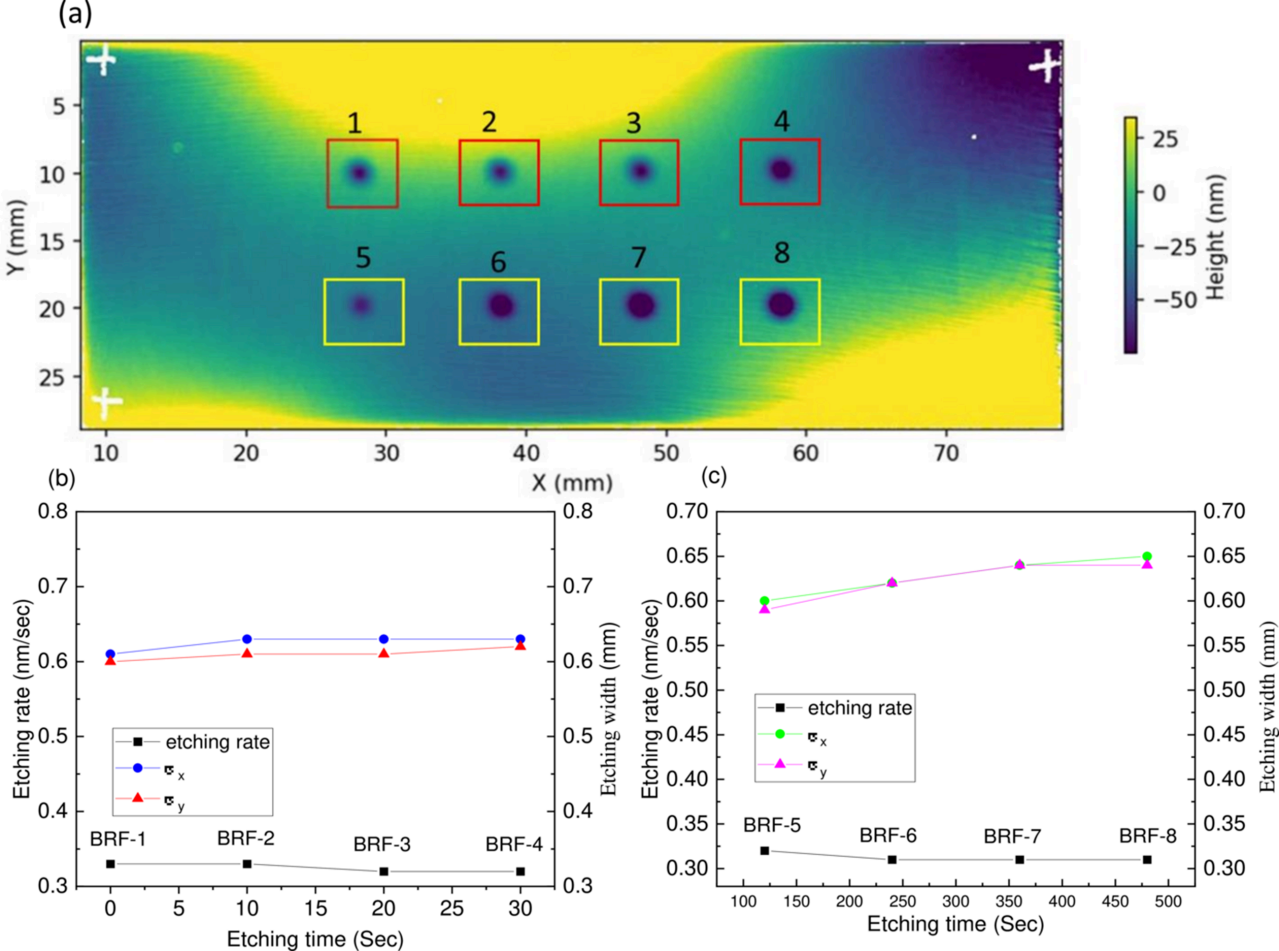
Temporal stability and determination of the removal rate of the ion beam at fixed parameters. (*a*) HDX Fizeau interferometry of the height profile of the Si test wafer to check the temporal stability of the ion beam. Two rows of etched footprints are visible. Footprints 1–4 (top row) were etched for the same length of time (240 s) and show consistent crater depths and widths. Footprints 5–8 (bottom row) were etched for increasing amounts of time (120 s, 240 s, 360 s and 480 s, respectively), and the craters increase in amplitude with etching time. (*b*) Analysis of removal rate and beam widths for footprints 1–4. Both the removal rate and the widths σ_*x*_ and σ_*y*_ remain constant with time, showing the ion beam has good temporal stability. (*c*) Analysis of the removal rate and beam width for footprints 5–8. The removal rate remains constant at around 0.32 nm s^−1^, showing that the removal from the ion beam is very linear. The beam width slightly increases, but generally remains stable at around σ_*x*_ = 0.63 mm and σ_*y*_ = 0.61 mm, confirming the linearity of removal during the ion beam figuring process.

To check the stability of the BRF function over time, two sets of beam footprints were etched onto a Si test wafer, using a circular aperture with a 1 mm diameter. Fig. 6 ▸(*a*) shows the measured height profile of the test wafer, including the beam footprints. The first set of footprints [top row, labelled 1–4 in Fig. 6 ▸(*a*)] were made to test the temporal stability of the ion beam. Each footprint was spaced 10 mm apart on the sample, etched for a fixed duration of 240 s, with a 10 min gap between successive etchings. The removal rate and beam width for each footprint were then obtained by fitting a 2D Gaussian function to the measured data. The removal rate and the beam widths σ_*x*_ and σ_*y*_ remained approximately constant with time, with an average removal rate of 0.33 nm s^−1^, and average beam widths σ_*x*_ = 0.63 mm and σ_*y*_ = 0.61 mm. This showed that the ion beam has good stability over time.

The second set of footprints [bottom row, labelled 5–8 in Fig. 6 ▸(*a*)] was made to determine the linearity of the removal rate for the beam. Each footprint was etched for a different duration, from 120 s to 480 s. Unlike the first set, there was no waiting time between footprints. As before, the removal rate and beam widths were obtained by fitting each crater to a 2D Gaussian function. Fig. 6 ▸(*c*) shows the removal rate and beam widths as a function of the time each crater was etched for. The removal rate is seen to be very stable with etching time, at a consistent ∼0.32 nm s^−1^. The beam width was also generally stable, with mean values of σ_*x*_ = 0.63 mm and σ_*y*_ = 0.61 mm, respectively.

## 4. RIFTA simulation

In a CCOS process, such as IBF, the user must calculate the required motion of the tool to correct a given surface profile. For a 2D surface in *x* and *y*, the removal at a given point*Z*(*x*_*i*_,*y*_*i*_) can be expressed as a convolution between the tool influence function TIF(*x*,*y*), representing the material removed by the polishing tool (here, an ion beam) per unit time, and *t*(*x*_*i*_,*y*_*i*_), the dwell time of the tool at that point,
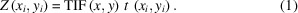
For IBF, *Z* is the height error profile measured across the optical surface, and TIF(*x*,*y*) is the beam removal function *B*(*x*,*y*), which may be determined experimentally as described in Section 5. The dwell time *t*(*x*,*y*) is then the unknown in this equation, which must be obtained in order to perform the figuring.

In principle, *t*(*x*,*y*) can be obtained by simply inverting equation (1) and solving the resulting deconvolution. However, solving such a deconvolution problem is not straightforward. It is generally ill-posed, having several possible solutions, some of which are unphysical, such as letting *t* < 0. In addition, there is often a trade-off between minimizing the residuals (that is, the difference between the actual surface height *Z* and the optimized removal *Z*_remov_), whilst also minimizing the total dwell time ∑*t*, to prevent prohibitively long duration figuring runs.

Many algorithms exist for solving equation (1) which satisfy non-negativity whilst minimizing the residuals and the total dwell time ∑*t*. Techniques involving inverse Fourier transforms are commonly used (Wilson & McNeil, 1987 ▸) as they are fast and computationally inexpensive. With this method, *t* can be obtained as
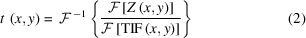
where 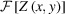 and 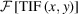 are the Fourier transforms of *Z* and TIF, respectively. However, this approach presents some issues regarding stability (such as when 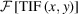 is close to zero), and care must be taken when designing an algorithm to avoid this problem.

This work utilizes one such algorithm, called RIFTA (robust iterative Fourier transform-based dwell time algorithm) (Wang *et al.*, 2020 ▸), developed at the National Synchrotron Light Source II (NSLS-II) in Brookhaven, USA. It employs a two-level iterative scheme to minimize the total dwell time, whilst ensuring non-negativity. The algorithm also automatically optimizes for the inverse filtering parameters, such that no additional hyperparameters must be set, and the process is a robust ‘out of the box’ solution.

**Figure 7:**
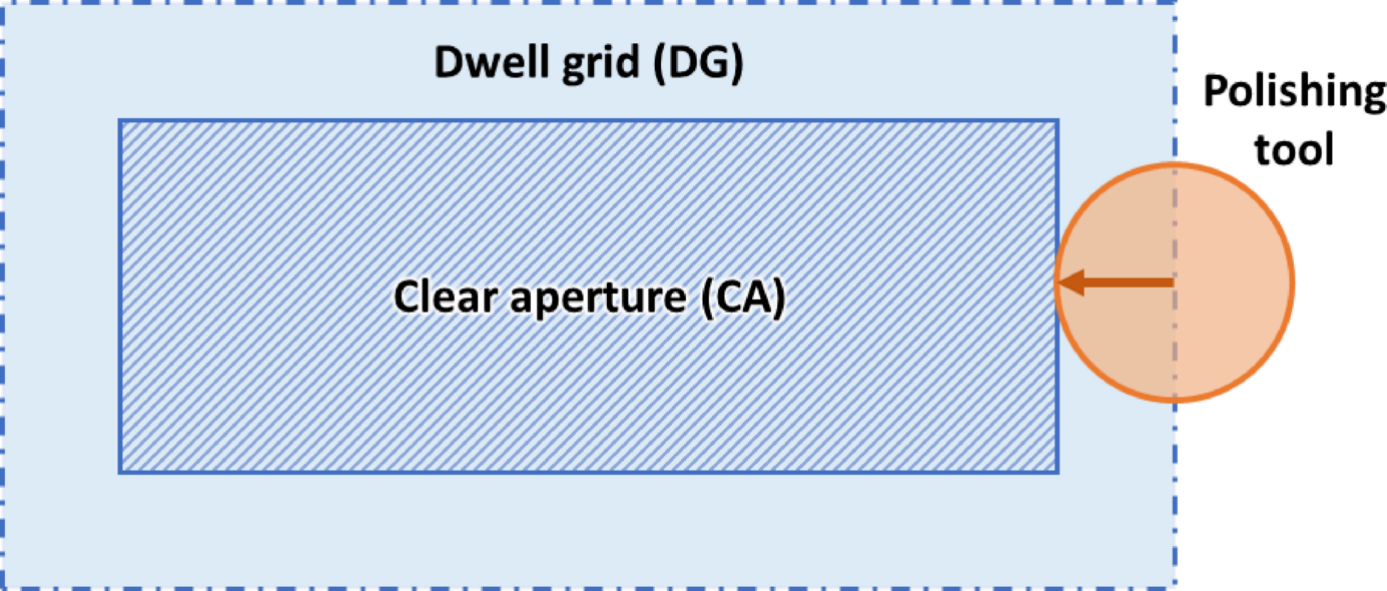
A schematic showing the clear aperture (CA), the area of the mirror over which correction is required. The dwell grid (DG), which is larger than the CA by the radius of the tool, is the area over which corrections are performed to avoid edge effects of the CA.

In a CCOS process, only performing the correction over the desired working region (called the clear aperture, or CA) will lead to edge effects, as parts of the surface near the edges are worked unevenly by the tool. Therefore, the correction is usually performed over an extended area called the dwell grid (DG), which is larger than the CA by the radius of the polishing tool to avoid edge effects (see Fig. 7 ▸ for a schematic). Therefore, when solving the deconvolution to obtain the dwell time map *t*(*x*,*y*), an extended surface height profile *Z*(*x*,*y*) must be used which covers the entire area of the dwell grid.

Whilst *Z* within the CA represents the measured surface height profile, the topography data in the outer region of the DG can be measured or artificial, as this will not impact the final performance of the optic. Indeed, using the measured height of the sample to populate the DG is generally unsuitable, as after one or more figuring runs the physical height outside the CA will be much lower than within the CA, due to characteristic ‘furrows’ left by the tool. Typically, surface extension algorithms are used to extrapolate the height within the CA to populate the DG, whilst minimizing edge effects and the total dwell time.

**Figure 8:**
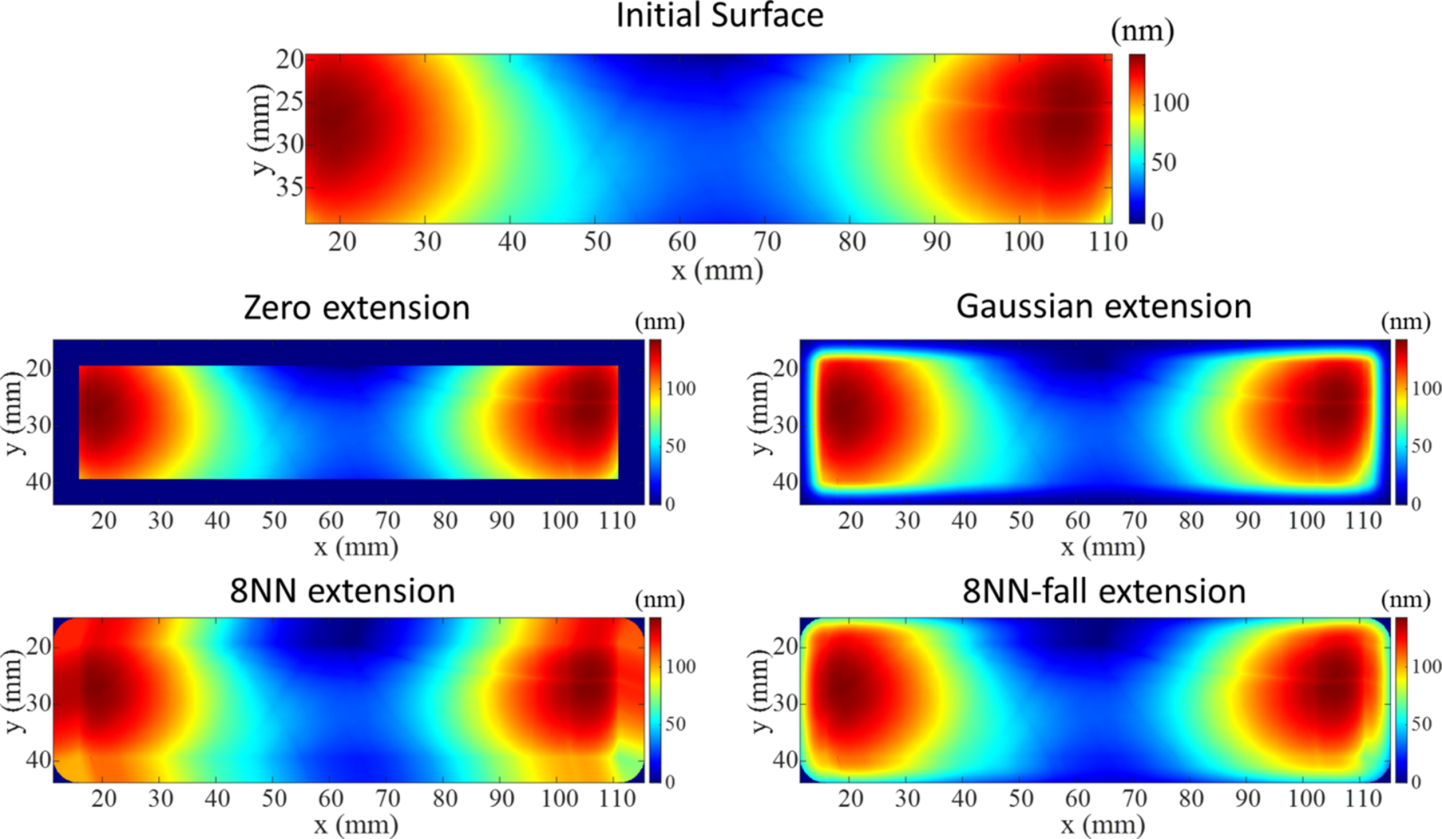
The results of extrapolating a 95 mm × 20 mm CA to a 105 mm × 30 mm DG using four different surface extension algorithms. In each case, the central region is filled with the data from the CA, and the margins around it are populated using different functions.

An analytical function is often used to extrapolate the measured data within the CA to the DG. This work employed four different surface extension algorithms (Wang, Huang *et al.*, 2021 ▸), from a library authored by T. Wang (https://github.com/TWANG006/surface-extension/tree/v1.1). Each extension type uses a different function to extrapolate the existing data. The zero extension populates the dwell grid with zeros; the Gaussian extension extrapolates measured data using a 1D Gaussian at each point on the boundary of the CA; and the eight-nearest-neighbour (8NN) extension uses nearest-neighbour extrapolation (Jiao *et al.*, 2009 ▸; Wu *et al.*, 2009 ▸). A multiplicative Gaussian fall-off term may also be used in the 8NN extension, which is then called the 8NN-fall extension. A schematic of the four surface extensions is shown in Fig. 8 ▸. The appropriate extension type for a given surface was chosen by assessing the calculated dwell time and residuals obtained with each extension.

**Figure 9:**
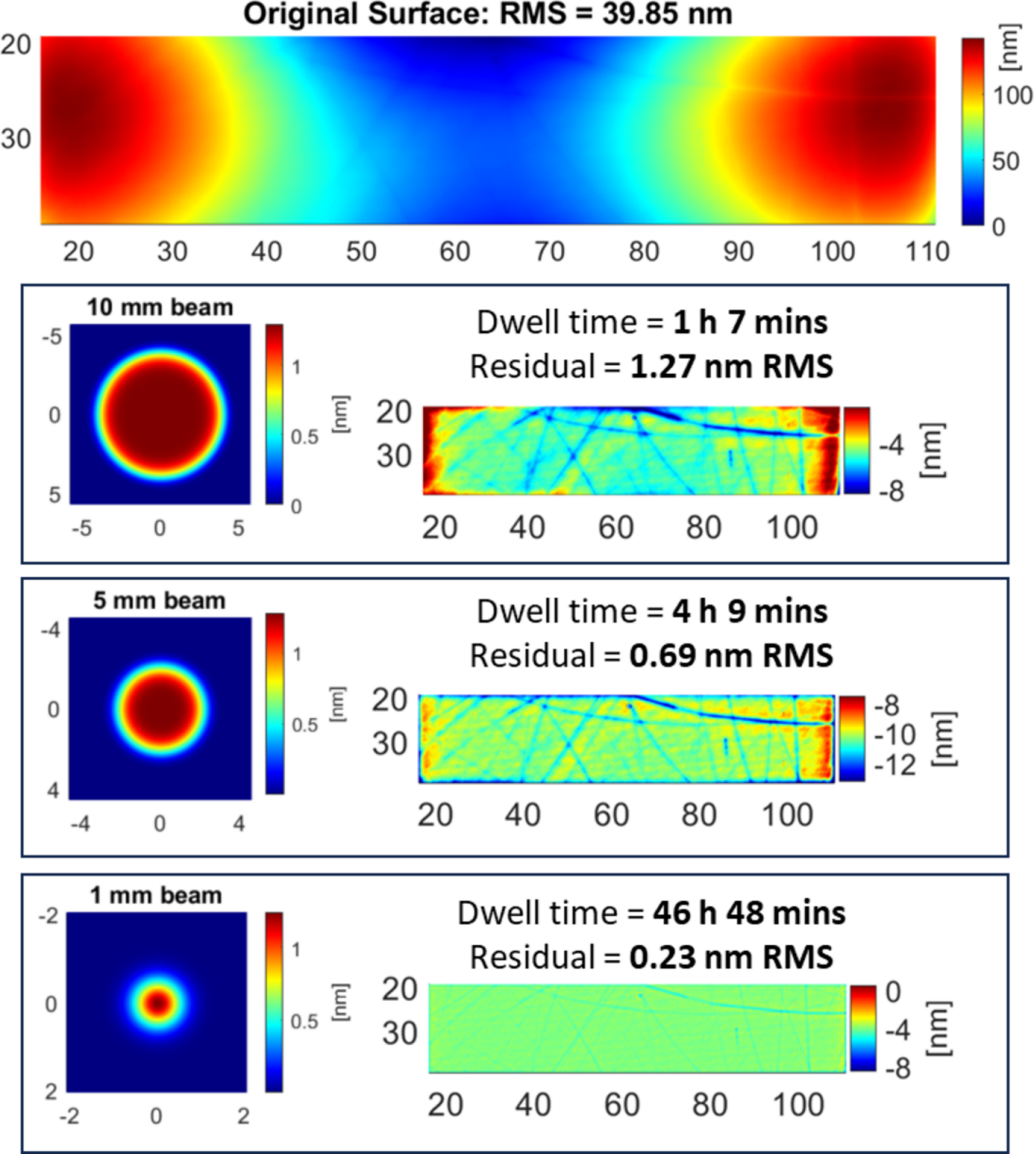
Calculated dwell time and residuals for IBF correction of a measured surface using a 10 mm, 5 mm and 1 mm beam. The initial surface is measured from the trapezoidal mirror in Section 5.2 (with a slope filter applied to remove spikes). The 10 mm beam is effective at minimizing the dwell time to around an hour, but the remaining residuals are comparatively large. The 5 mm beam gives improved residual errors, but with an increased dwell time. Finally, the 1 mm beam minimizes the residual errors, but requires a prohibitively long dwell time of almost 47 h.

The optimal beam shape and size to correct each surface was also assessed using the results of RIFTA calculations. Fig. 9 ▸ shows the calculated dwell time and residuals for a test surface when using a 1 mm, 5 mm or 10 mm beam. A larger beam size is suitable for correcting coarse errors with longer spatial periods, whereas a smaller beam size is suitable for fine error correction. Depending on the surface, IBF is generally performed in several iterations, beginning with larger beam sizes and progressing to figuring using smaller beam sizes until the desired figure accuracy is achieved.

## 5. Results and discussions

### 5.1. 1D ion beam figuring

1D correction of a Si X-ray mirror was performed using IBF over a 50 mm line. A rectangular aperture of 0.5 mm × 8 mm (horizontal × vertical) was used to shape the ion beam profile. To calculate dwell time for the 1D correction, a combination of RIFTA with a matrix-based method was used, which utilizes least-squares minimization to solve the deconvolution (Carnal *et al.*, 1992 ▸). Such matrix-based methods require a long computation time in the 2D case but are fast and flexible for 1D calculations. The height profile of the mirror before and after correction was measured using stitching interferometry, and the slope error was calculated from the height data with a 1.5 mm low-pass filter applied to remove the high-frequency components. This filtering step is essential because high-frequency features can dominate, resulting in an r.m.s. error to a higher level. Since the IBF system is not able to correct such high frequency details on the mentioned surface, a band-limited r.m.s. slope error is calculated to have a proper metric to compare the corrections made by the IBF system.

**Figure 10:**
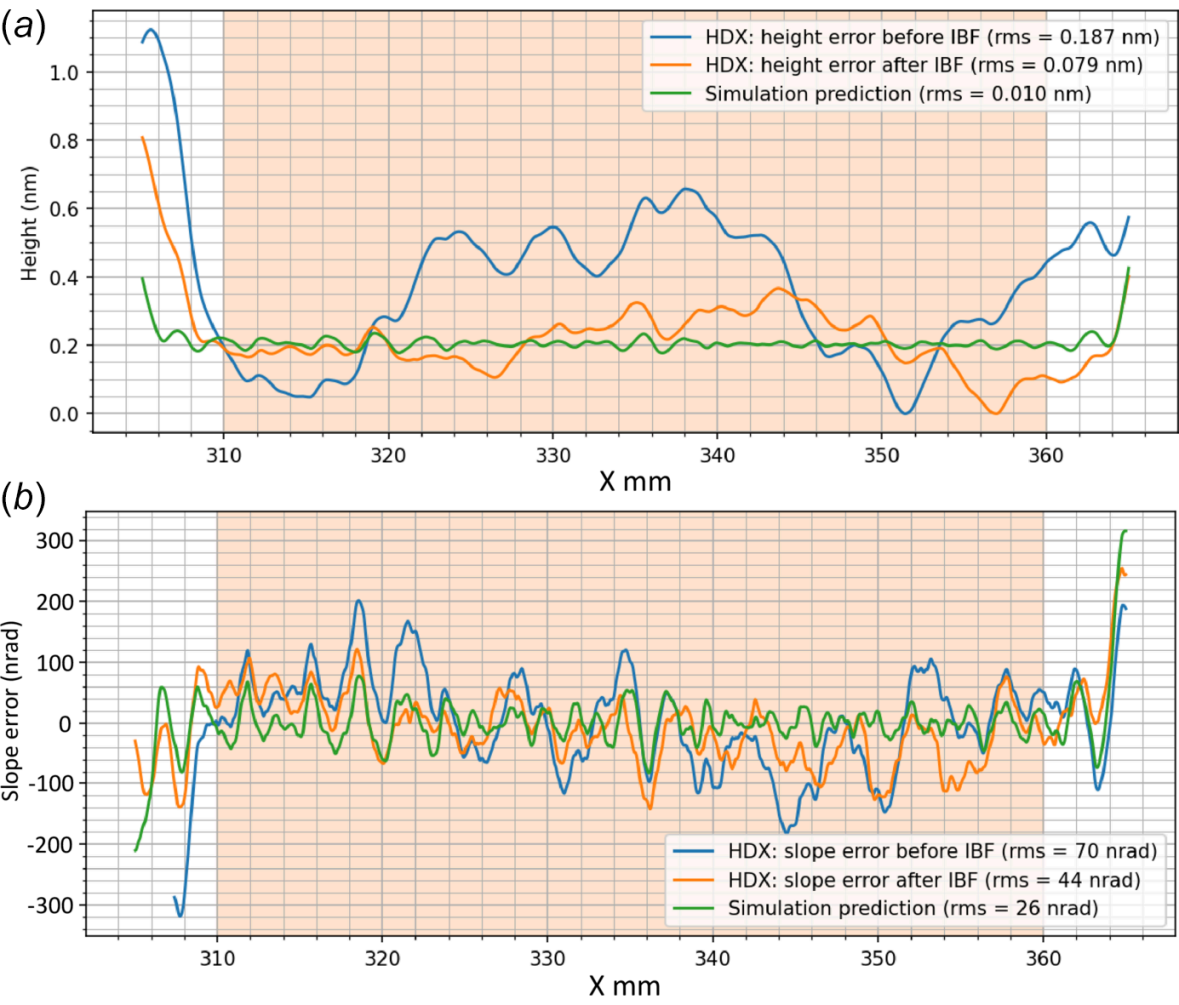
Fizeau interferometry measurements of an Si mirror before (blue curves) and after (orange curves) 1D-IBF correction, showing improvements in (*a*) height error and (*b*) slope error. In both cases, the residuals are significantly reduced by the correction process, which is converging towards the simulated predictions (green curves).

Fig. 10 ▸ shows the results of the 1D IBF of the test mirror for correcting the height error (*a*) and slope error (*b*). The initial figure errors of the mirror were already low, with an initial height error of 0.187 nm r.m.s. and a slope error of 70 nrad r.m.s. Using IBF, the figure errors were improved even further, to a height error of 0.079 nm r.m.s. and a slope error of 44 nm r.m.s. This represents a reduction by more than a factor of 2 in height error and more than a factor of 1.5 in slope error. The residual height and slope errors also show good agreement with the predicted values obtained from simulation, although the real residual errors are slightly higher than the predicted values (0.01 nm for the height error, and 26 nrad for the slope error). Overall, the 1D figuring results confirmed a good agreement between simulated predictions and experimental outcome for the IBF process.

While limited in scope, 1D correction can prove adequate for certain X-ray beamline mirrors. Take, for instance, focusing Kirkpatrick–Baez (KB) mirrors, which primarily focus on a narrow central region, operating within a specific perspective. In the correction process for such mirrors, typically only this central region is taken into account, making 1D correction satisfactory to attain optimal performance (Morawe *et al.*, 2019 ▸; Zhou *et al.*, 2016 ▸)

### 5.2. 2D ion beam figuring

**Figure 11:**
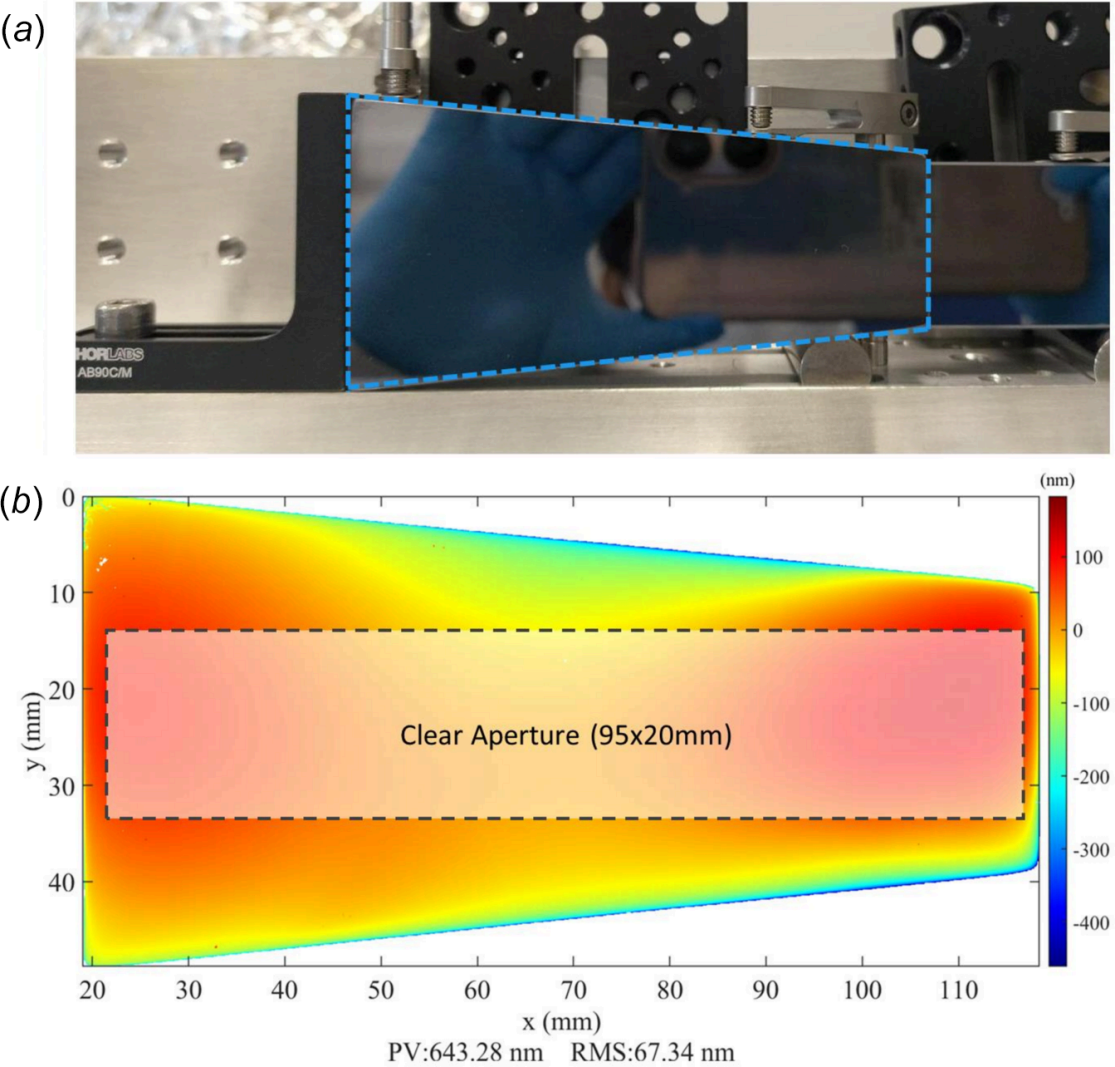
(*a*) Photograph of the trapezoidal silicon mirror used for 2D-IBF correction (dashed outline), mounted on a support plate in preparation for loading into the IBF chamber. (*b*) HDX measurement of the initial height profile of the mirror, with the selected clear aperture region shown (dashed outline).

**Figure 12:**
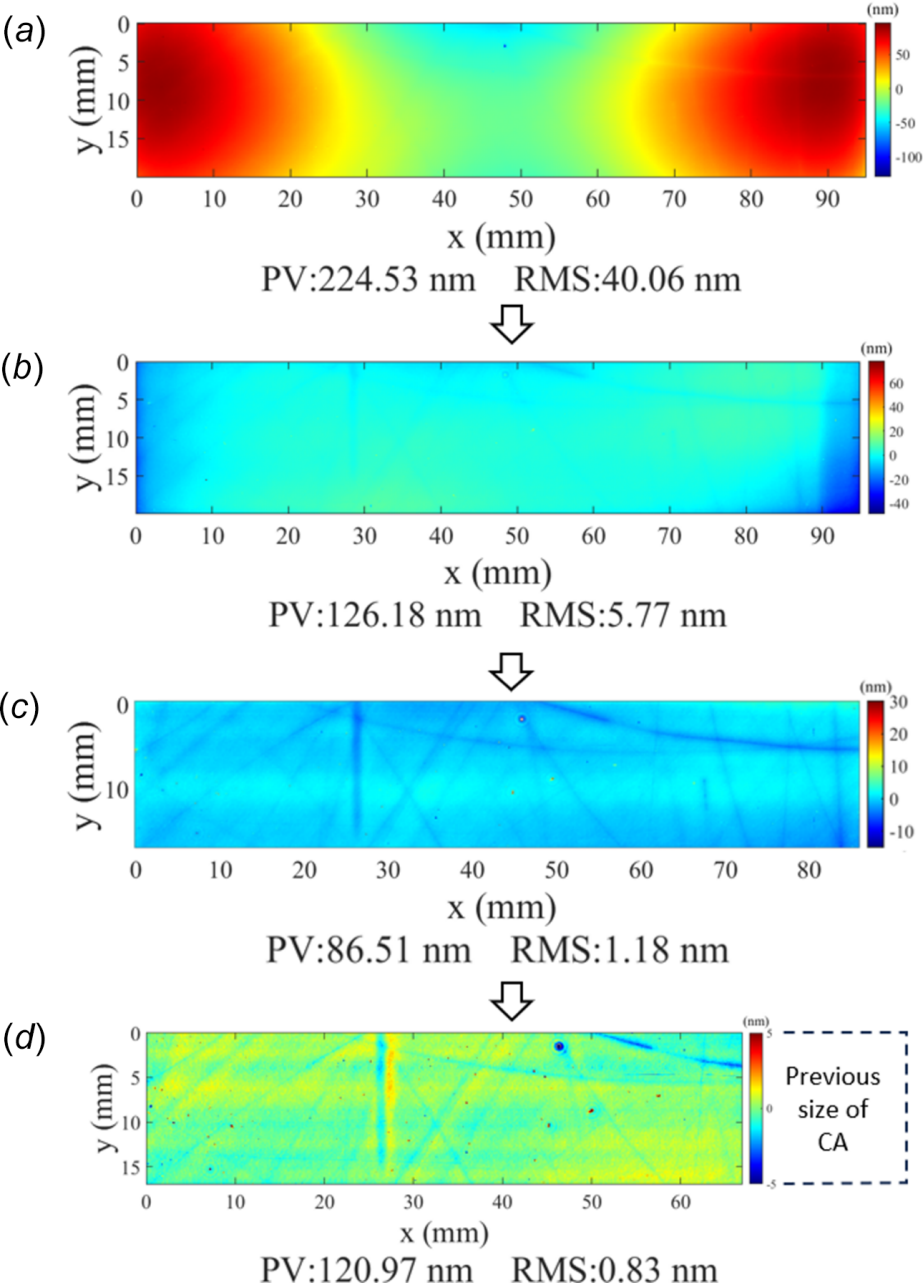
Results of the 2D-IBF process on a 95 mm × 20 mm clear aperture within a trapezoidal Si mirror. (*a*) Initial measured height of the mirror over a 95 mm × 20 mm clear aperture, showing low-frequency height errors of several hundred nm amplitude. (*b*) The measured height after one iteration of IBF using a 5 mm beam. The PV and r.m.s. height errors are both significantly reduced. (*c*) The measured height after a second iteration of IBF with a 5 mm beam, showing further reduction in the r.m.s. height error. (*d*) The measured height after the third and final iteration of IBF, using a 1 mm beam. The r.m.s. height errors converge to below 1 nm, showing excellent performance of the figuring. The high residual PV is due to isolated spikes on the surface, which represent pits or scratches that were present on the surface before the IBF process.

Following the validation of the 1D-IBF process, 2D correction was performed using IBF on an Si mirror. The mirror was a refurbished optic that had previously been deployed on a beamline at Diamond, and its reflective coating was chemically stripped before the IBF process. The mirror had a trapezoidal shape, measuring approximately 100 mm in length, 50 mm wide on its long edge, and 30 mm wide on its short edge, as shown in Fig. 11 ▸(*a*). A clear aperture of 95 mm × 20 mm was selected for figuring, shown in Fig. 11 ▸(*b*). The initial surface within the CA had height errors of 224 nm peak-to-valley (PV) and 40 nm r.m.s. [Fig. 12 ▸(*a*)].

The first iteration of IBF was performed using a 5 mm beam aperture, with a total dwell time of around 7 h. After this first iteration, the residual height error was reduced to 126 nm PV and 5.7 nm r.m.s., as shown in Fig. 12 ▸(*b*). This represents a reduction of around a factor of 2 in the PV errors, and a reduction of a factor of 7 in the r.m.s. errors, showing that the surface was greatly improved after only one iteration of IBF. Additionally, these results show good convergence to the predicted results from simulation of 106 nm PV and 0.95 nm r.m.s.

The errors were then further reduced by performing two additional iterations of IBF, using first a 5 mm beam and then a 1 mm beam. The residual height error was improved to 86.5 nm PV, 1.18 nm r.m.s. by the second iteration [Fig. 12 ▸(*c*)], and was reduced to a final value of 120 nm PV and 0.8 nm r.m.s. by the third iteration [Fig. 12 ▸(*d*)]. The final CA was reduced to 67 mm × 17 mm, due to accidental over-etching of the rightmost part of the CA during the third iteration caused by unexpected ion flux outside of the beam centre. These results demonstrate rapid convergence to sub-nanometre r.m.s. height errors in only three iterations of the IBF process. The total machining time for the three iterations is calculated as follows: 7 h for the first iteration, 2.14 h for the second iteration and 4.92 h for the third iteration, resulting in a cumulative time of 14 h. The tangential slope error before and after IBF was calculated from the measured height data over a 3 mm-wide line, with a 2.5 mm low-pass filter applied to remove high-frequency components from the data. For the initial surface, the slope error was 1.30 µrad r.m.s., which was reduced to 230 nrad r.m.s. after the third figuring iteration. As with the height error, this shows that the slope error can be rapidly and efficiently reduced by only a few iterations of the IBF process.

A fourth iteration was attempted, but the results showed that the r.m.s. errors could not be further improved by the IBF process. This is likely due to the scratches and grooves present on the surface of this sample, which are visible in Fig. 12 ▸(*d*).

While satisfactory convergence has been achieved in every iteration, deviations from predictions offered by the RIFTA model may occur due to various sources of error inherent in real-world machining processes and experimental conditions. These include discrepancies in removal function derivation, approximation errors in dwell time conversion to a smooth position–velocity–time distribution, inaccuracies in positioning and temperature fluctuations inducing stress deformation. To minimize thermal fluctuations, precise surface metrology is conducted after optics have completely cooled for several hours.

### 5.3. Impact of IBF on micro-roughness

**Figure 13:**
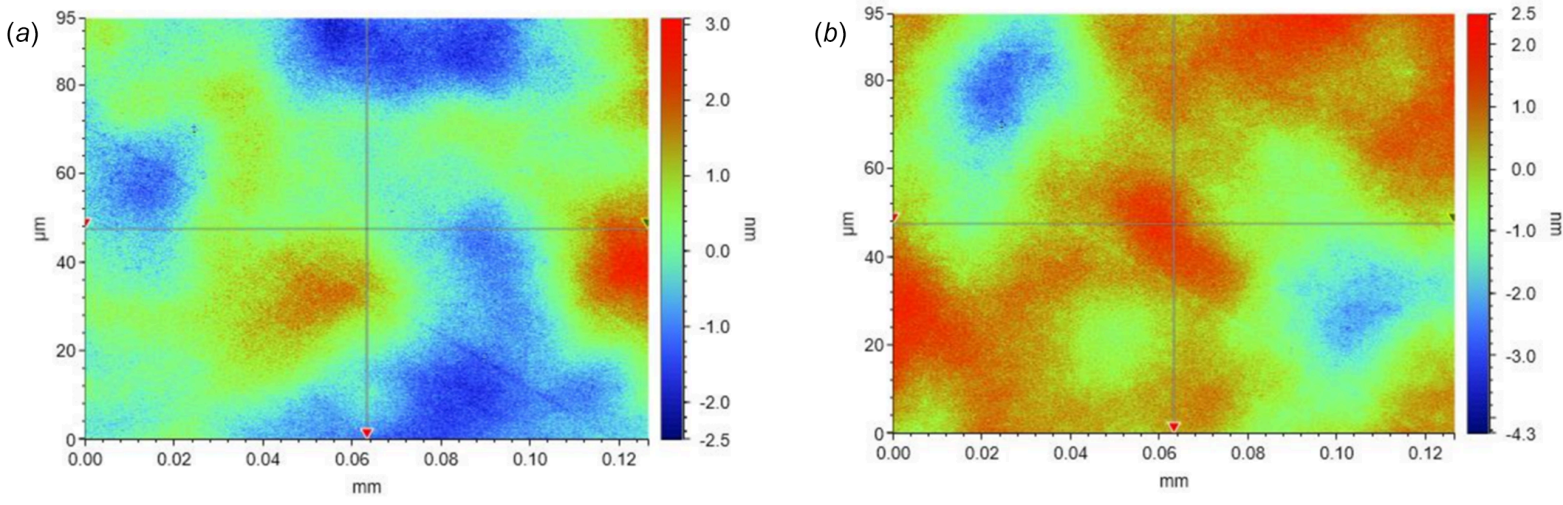
2D height profiles of the trapezoidal mirror before (*a*) and after (*b*) IBF, measured using GTX micro-interferometry at 50× magnification. The micrometre-scale features of the sample are similar before and after IBF, with the micro-roughness increasingly slightly from 0.82 to 0.92 nm r.m.s.

To ensure good optical performance of X-ray optics, it is important that the IBF process does not increase the surface micro-roughness. To verify this, the micro-roughness of a Si mirror was measured using a GTX micro-interferometer before and after IBF processing, which allows micrometre-scale features to be measured with a spatial resolution of 0.2 µm. Fig. 13 ▸ shows micro-interferometry images of the optic before and after IBF. The general surface characteristics are similar in each case. The 2D micro-roughness was increased slightly from 0.82 to 0.92 nm r.m.s. Whilst these results are encouraging, further work is required to determine whether the micro-roughness of ultra-smooth optics (<0.3 nm r.m.s.) is worsened by IBF processing.

## 6. Conclusions

1D and 2D correction of Si X-ray mirrors has been demonstrated using the ion beam figuring system developed at Diamond Light Source. The results show improvement of height and slope errors in both 1D and 2D, with the 1D figuring yielding r.m.s. height errors below 0.1 nm over a 50 mm region, and the 2D figuring producing r.m.s. height errors below 1 nm in a 67 mm × 17 mm area. The 1D and 2D figuring results also showed close agreement with the predicted results from simulations, which indicates that the figuring process is stable and well controlled.

Achieving sub-nanometre r.m.s. height errors is a crucial milestone for high-quality X-ray optics. These results from the Diamond IBF project are approaching state-of-the-art quality, which is highly encouraging. Future work will focus on 2D correction of optics, with the goal of reducing slope error below 100 nrad r.m.s. alongside sub-nanometre r.m.s. height errors. It is hoped that the IBF facility at Diamond will soon be able to deliver beamline-quality optics.
